# Ovarian Reserve after Chemotherapy in Breast Cancer: A Systematic Review and Meta-Analysis

**DOI:** 10.3390/jpm11080704

**Published:** 2021-07-23

**Authors:** Alessia Romito, Sonia Bove, Ilaria Romito, Drieda Zace, Ivano Raimondo, Simona Maria Fragomeni, Pierluigi Maria Rinaldi, Domenico Pagliara, Antonella Lai, Fabio Marazzi, Claudia Marchetti, Ida Paris, Gianluca Franceschini, Riccardo Masetti, Giovanni Scambia, Alessandra Fabi, Giorgia Garganese

**Affiliations:** 1 Gynecology and Breast Care Center, Mater Olbia Hospital, 07026 Olbia, Italy; alessia.romito@materolbia.com (A.R.); sonia.bove@materolbia.com (S.B.); ivano.raimondo@materolbia.com (I.R.); domenico.pagliara@materolbia.com (D.P.); giovanni.scambia@policlinicogemelli.it (G.S.); giorgia.garganese@materolbia.com (G.G.); 2 Dipartimento Scienze della Vita e Sanità Pubblica, Università Cattolica del Sacro Cuore, 00168 Roma, Italy; drieda.zace@unicatt.it (D.Z.); claudia.marchetti@policlinicogemelli.it (C.M.); 3 Dipartimento Scienze della Salute della Donna, del Bambino e di Sanità Pubblica, Fondazione Policlinico Universitario Agostino Gemelli IRCCS, 00168 Roma, Italy; simona.fragomeni@policlinicogemelli.it (S.M.F.); ida.paris@policlinicogemelli.it (I.P.); gianluca.franceschini@policlinicogemelli.it (G.F.); riccardo.masetti@policlinicogemelli.it (R.M.); alessandra.fabi@policlinicogemelli.it (A.F.); 4 Radiology and Interventional Radiology Unit, Mater Olbia Hospital, 07026 Olbia, Italy; pierluigi.rinaldi@materolbia.com; 5 Dipartimento di Diagnostica per Immagini, Radioterapia Oncologica ed Ematologia, Fondazione Policlinico Universitario Agostino Gemelli IRCCS, 00168 Roma, Italy; fabio.marazzi@unicatt.it; 6 Department of Oncology, Mater Olbia Hospital, 07026 Olbia, Italy; antonella.lai@materolbia.com; 7 Dipartimento di Medicina e Chirurgia traslazionale, Università Cattolica del Sacro Cuore, 00168 Roma, Italy

**Keywords:** breast cancer, AMH, ovarian reserve, chemotherapy, pregnancy desire

## Abstract

**Background**: Worldwide, breast cancer (BC) is the most common malignancy in the female population. In recent years, its diagnosis in young women has increased, together with a growing desire to become pregnant later in life. Although there is evidence about the detrimental effect of chemotherapy (CT) on the menses cycle, a practical tool to measure ovarian reserve is still missing. Recently, anti-Mullerian hormone (AMH) has been considered a good surrogate for ovarian reserve. The main objective of this paper is to evaluate the effect of CT on AMH value. Methods: A systematic review and meta-analysis were conducted on the PubMed and Scopus electronic databases on articles retrieved from inception until February 2021. Trials evaluating ovarian reserves before and after CT in BC were included. We excluded case reports, case-series with fewer than ten patients, reviews (narrative or systematic), communications and perspectives. Studies in languages other than English or with polycystic ovarian syndrome (PCOS) patients were also excluded. AMH reduction was the main endpoint. Egger’s and Begg’s tests were used to assess the risk of publication bias. Results: Eighteen trials were included from the 833 examined. A statistically significant decline in serum AMH concentration was found after CT, persisting even after years, with an overall reduction of −1.97 (95% CI: −3.12, −0.82). No significant differences in ovarian reserve loss were found in the BRCA1/2 mutation carriers compared to wild-type patients. Conclusions: Although this study has some limitations, including publication bias, failure to stratify the results by some important factors and low to medium quality of the studies included, this metanalysis demonstrates that the level of AMH markedly falls after CT in BC patients, corresponding to a reduction in ovarian reserve. These findings should be routinely discussed during oncofertility counseling and used to guide fertility preservation choices in young women before starting treatment.

## 1. Introduction

Breast cancer (BC) is the most common cancer in the female population, with an estimated 2.3 million new cases worldwide in 2020 [1]. 

While the death rate has dropped by 40% since 1989, an increase in BC among young women has been reported, with around 10% of new cases diagnosed in patients younger than 40 years old [2]. 

Physicians should carry out counseling on fertility issues and fertility preservation in patients who have not completed childbearing before starting treatment [3].

Recent evidence has shown that age and the use of cyclophosphamide-based chemotherapy are the main factors influencing ovarian failure. In particular, the risk of chemotherapy-induced amenorrhea (CIA) was 55% [95% CI 50–60%], ranging from 26% to 77% in women younger than 35 years old compared to those older than 40 [4]. ]. However, CIA might not be the best indicator of fertility because resumption of menses can resume even one year after chemotherapy treatment [4]. In the last two decades, there has been increased interest in the role of anti-Mullerian hormone (AMH) as a marker of ovarian reserve. Preantral and antral follicles, which remain in the growing phase for many weeks, independently of FSH or menses fluctuation, release AMH [5]. AMH is superior in terms of accuracy compared to other conventional indicators of ovarian function such as menstruation, estradiol or FSH [6,7]. Recently, a growing body of literature has been published on the effect of chemotherapy on the ovarian reserve in women with BC, assessed by AMH [8,9,10,11,12,13,14,15,16,17,18,19,20,21,22,23,24,25]. The summarization and synthesis of these results could provide BC patients and healthcare professionals with crucial information on the reproductive health consequences of chemotherapy treatment and the possibility of achieving pregnancy after completion of endocrine therapy.

In this context, this systematic review aims to evaluate the impact of chemotherapy treatment on the ovarian reserve in fertile women with BC through the quantification of AMH.

## 2. Materials and Methods

The present work has been reported according to the Preferred Reporting Items for Systematic Reviews and Meta-Analyses (PRISMA) statement [26].

### 2.1. Research Question

To address our objective, we structured a specific research question based on the PI/ECOS framework (Population, Intervention/Exposure, Comparison, Outcome, Setting/Time) as follows:

- Population: Fertile women who have been diagnosed with breast cancer and have gone through chemotherapy treatment
- Intervention: Chemotherapy treatment
- Comparison: Not Applicable
- Outcome: Ovarian reserve measured by AMH
- Setting/Time: All

### 2.2. Literature Search

The research was conducted on the PubMed and Scopus electronic databases. A search string was first built for PubMed, using MeSH terms, Boolean operators, and free text words. The string was subsequently adapted for use in the other database. The search was restricted to articles published in English, without any further restrictions and was last performed on 17 February 2021 for all databases. Supplementary Material (Table S1) shows the complete search strategy.

The reference lists of included studies were hand-searched for additional articles. Reference lists with trials, previous reviews or meta-analyses were also reviewed.

### 2.3. Study Selection and Inclusion/Exclusion Criteria

All studies that compared AMH levels at baseline and after chemotherapy in BC patients younger than 50 years old were considered pertinent. Only studies that reported a minimum of one-month follow-up were included in the systematic review. Only peer-reviewed articles reporting primary data, with no time limits, were included. We excluded case reports, case-series (reporting data for fewer than ten patients) reviews (narrative or systematic), communications and perspectives. We also excluded studies in languages other than English or with polycystic ovarian syndrome (PCOS) patients.

All articles retrieved from the search strategy were imported to Rayyan QCRI [27] and duplicates were removed. Two independent reviewers (A.R. and I.R.) selected the identified studies based on the title and abstract. We evaluated the full-text version of the studies if the title or abstract did not clarify the topic.

Discussion by a multidisciplinary team, including breast surgeons (S.B., S.M.F. and D.P.), gynecologists (Iv.R. and C.M.), a radiologist (P.M.R.) and medical oncologists (A.L., F.M. and I.P.) resolved uncertainties about the eligibility of the papers. A statistician experienced in meta-analysis (D.Z.), along with the two reviewers, analyzed data. We discussed the results in a multidisciplinary setting.

Finally, an expert committee (G.F., R.M., G.S., A.F. and G.G.) performed an independent review and gave the final approval.

### 2.4. Data Extraction

Two researchers (A.R. and I.R.) performed the data extraction process. We used a standardized Excel spreadsheet to extract following data: first author; year of publication; study design; sample size; mean age of patients; type of chemotherapy; follow-up duration; AMH assay; basal AMH; AMH immediately after chemotherapy; and AMH at 6 months, 1 year, 2 years and 3 years of follow-up.

### 2.5. Data Synthesis and Statistical Analyses

Treatment effect, defined as the difference between basal AMH and the value of AMH immediately, 6 months, 1 year, 2 years and 3 years after treatment, was calculated for each included study. If the AMH was undetectable after chemotherapy, we used the lower detection limit for the specific assay. The variance in the treatment effect, derived from standard deviations or standard errors of paired differences between baseline and the end of follow-up, was calculated. If these statistics were not given, they were calculated, where appropriate data were available [28]. The mean effect size was calculated using the inverse variance method in random-effect models. Forest plots were used for the graphical representation of each study. Heterogeneity was examined using the I^2^ test, with I^2^ > 50% considered important [29]. Publication bias was examined using the analyses described by Egger and Begg, where *p* < 0.05 indicated significant publication bias [30,31]. Galbraith’s test and sensitivity analysis were conducted to investigate the impact each study had on the overall estimate and its contribution to Q-statistics [32]

An overall analysis of data from all studies was performed (at 1 month, 6 months, 1 year, 2 years and 3 years of follow-up). Subsequently, to account for confounding factors, subgroup analyses were performed based on the patient’s age, AMH kits used, BRCA status, chemotherapy regimen and hormone therapy. Statistical analysis was performed using Cochrane Collaboration RevMan 5.1 software (http://www.cochrane.org, accessed on 16 May 2021) and STATA software (StataCorp, 2015, Stata Statistical Software: Release 15; StataCorp LP 4905 Lakeway Drive, College Station, TX, USA).

### 2.6. Quality Assessment

The methodological quality of the included studies was assessed based on the study design. The Newcastle–Ottawa scale was applied for cohort studies to evaluate the following quality parameters: selection of study groups, comparability of study groups and ascertainment of outcome, giving scores that range from 0 to 9. The Jadad tool was used to assess the methodological quality of randomized controlled trials (RCTs) included in the systematic review. It evaluates the randomization process, blinding, dropouts and withdrawal and assigns up to 5 points. To summarize the overall evidence quality, we grouped the articles into three categories: good methodological quality (studies that met at least 75% of the quality criteria), moderate methodological quality (studies that met between 50% and 74% of the quality criteria) and poor methodological quality (studies that met less than 50% of the quality criteria).

## 3. Results

**Figure 1:**
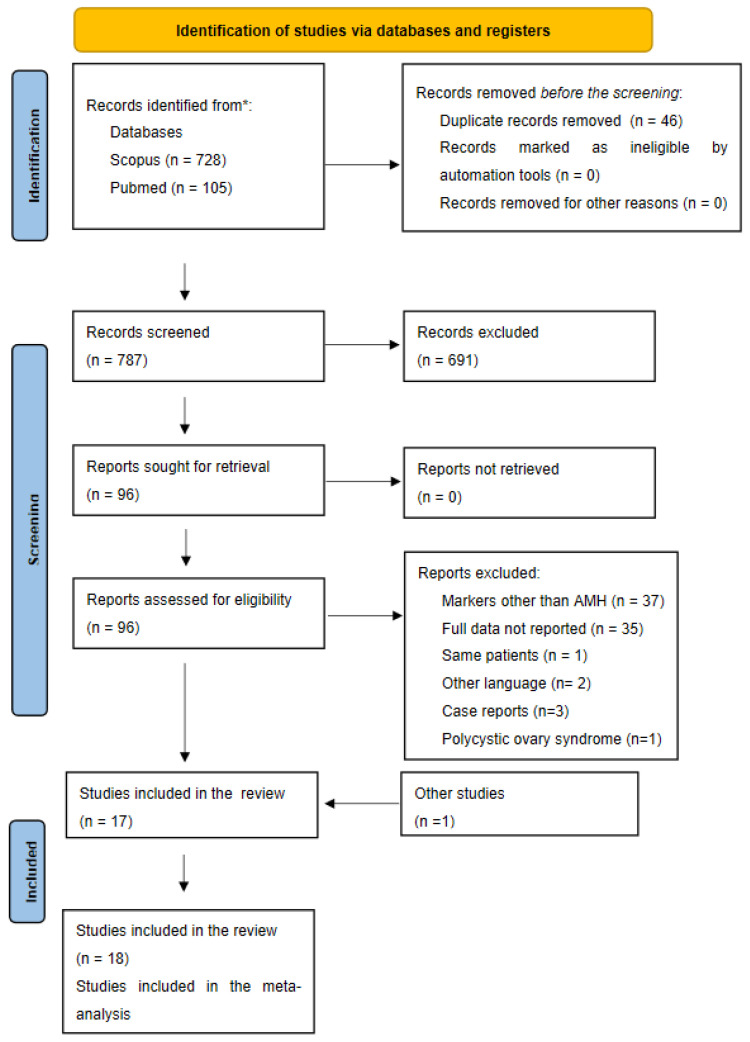
PRISMA flow diagram.

Figure 1 shows the number of studies assessed and excluded through the stages of the meta-analysis.

### 3.1. Bibliographical Search

The defined search strategy retrieved 833 studies from PubMed and Scopus. After the first screening process, 96 articles were deemed pertinent, and their full texts were thoroughly read. Based on this step, 79 articles were excluded for the following reasons: outcome other than AMH, incomplete data and full text in languages other than English. One study was excluded because it reported the same sample of patients as another [33] and three studies were excluded because they included fewer than ten patients [34,35,36,37]. One article was additionally added after hand-searching the references of the included studies [8]. A total of 18 studies were deemed eligible for this systematic review and meta-analysis [8,9,10,11,12,13,14,15,16,17,18,19,20,21,22,23,24,25]. Anderson et al. carried out two studies on the same population but with different follow-up periods; thus, both studies were included [10,11].

### 3.2. Description of the Included Studies

The main characteristics of the included studies are shown in Supplementary Material (Tables S2 and S3). Nine studies (50%) were carried out in Europe [10,11,15,16,18,21,22,23,24], three (16%) were carried out in the USA [17,20,25], four (22%) were carried out in Asia [8,9,12,19], one (6%) was carried out in Africa [14] and one (6%) was carried out in South America [13].

All included studies had a cohort design [8,9,10,11,13,14,15,17,18,19,20,21,22,23], except for three RCT [16,24,25] and one case–control study [12]. The study by Trapp et al. was a sub-analysis of the SUCCESS-A study [37]. It compared the disease-free survival (DFS) of patients receiving three cycles of fluorouracil, epirubicin and cyclophosphamide (FEC) followed by three cycles of docetaxel (D) versus three cycles of FEC chemotherapy followed by three cycles of gemcitabine and docetaxel (DG). This study included only premenopausal patients, younger than 40 years old, treated with FEC-D. The RCT of Hadji et al. and Yu et al. randomized patients to receive zoledronic acid versus a placebo after the standard treatment. For both studies, the two aims were combined in the overall analysis [16,25].

The sample size varied from 23 [13] to 250 patients [15], for a total of 1219 women. The mean age varied from 26 [13] to 41 [10,11]. The chemotherapy regimen was variable (cyclophosphamide, FEC, FEC-D, FEC-D plus methotrexate plus fluorouracil (CFM), doxorubicin plus cyclophosphamide (AC) in association with taxanes (AC-T), treatment with aGnRH during or after chemotherapy, and hormone therapy often not reported (in 52%, 32% and 37% of the studies, respectively). Six different AMH kits were used. Elecsys AMH assay was used in four studies [13,18,21,22], AMH Gen II assay was used in six studies [8,12,16,17,19,24], four articles used the Immunotech (IOT) [10,11,14,15], one article used the Diagnostic Systems Laboratories (DSL) [25], one used the ultrasensitive AMH ELISA kit [23] and one used the picoAMH ELISA kit [20]. One study did not specify the AMH kits used [9].

Seven studies reported AMH value immediately after chemotherapy [8,9,14,17,21,24,25], four studies reported it six months after chemotherapy [10,14,16,25], ten studies reported it one year after chemotherapy [10,13,16,17,18,19,20,22,23,25], four studies reported it two years after chemotherapy [11,15,20,24] and three studies reported it three years after chemotherapy [11,18,22].

### 3.3. Quality Assessment

Five out of fifteen cohort and case–control studies were considered of high quality (satisfied 75% or more of the quality criteria), while two out of three RCT were of medium quality (satisfied between 50% and 74% of the quality criteria) (Supplementary Material: Tables S4 and S5).

### 3.4. Meta-Analysis

All studies were analyzed, followed by a subgroup analysis for age, AMH kits and BRCA status. The insufficient data did not permit the subgroup analysis for a chemotherapy regimen and endocrine therapy.

#### 3.4.1. Ovarian Reserve after Chemotherapy

**Figure 2:**
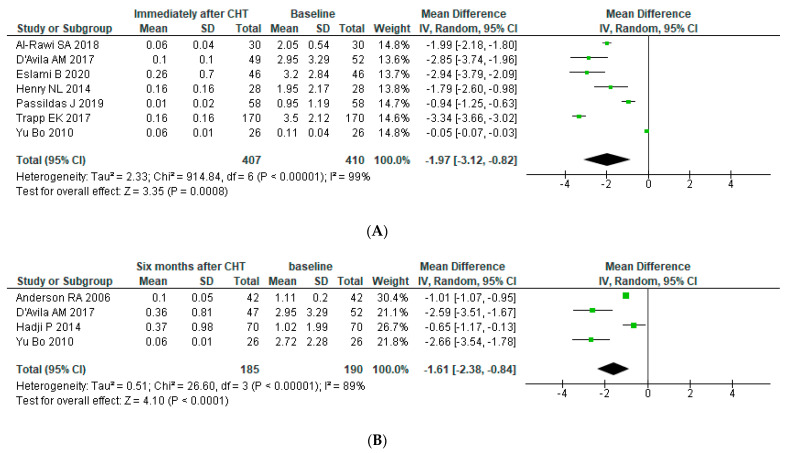

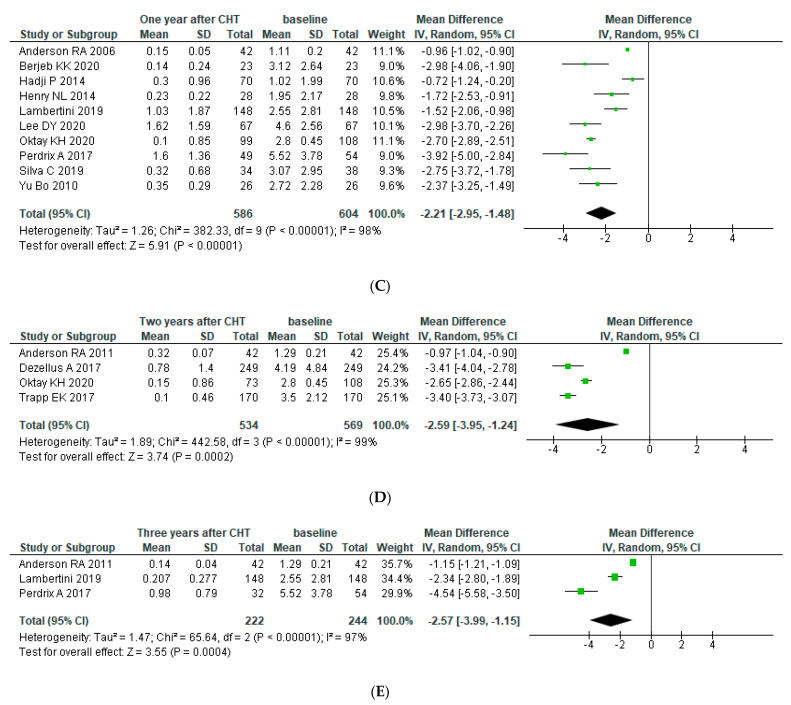
Forest plot for post-operative AMH levels in all BC women. AMH level were assessed (**A**) immediately after chemotherapy, (**B**) 6 months later, (**C**) 1 year later, (**D**) 2 years later and (**E**) 3 years later.

An analysis of seven studies including 410 patients revealed a statistically significant decline in serum AMH concentration immediately after chemotherapy, with an overall reduction of −1.97 (95% CI: −3.12, −0.82). There was significant heterogeneity in the pooled analysis (*p* for heterogeneity < 0.00001, I2 = 99%). After six months, there was a slight recovery (Mean Difference (MD): −1.61 (95% CI: −2.38, −0.84)) with a pooled population of 190 patients (*p* for heterogeneity < 0.00001, I2 = 89%). One year after chemotherapy, there was a further reduction (MD: −2.21 (95% CI: −2.95, −1.48)) (*p* for heterogeneity < 0.00001, I2 = 98%) to achieve a steady state after two and three years of follow-up (MD after 2 and 3 years: −2.59 (95% CI: −3.95, −1.24) (*p* for heterogeneity < 0.00001, I2 = 99%) and −2.57 (95% CI: −3.99, −1.15) (*p* for heterogeneity < 0.00001, I2 = 97%), respectively (See Figure 2)). Galbraith’s test was conducted, but heterogeneity remained high (more than 80%). Egger’s and Begg’s tests showed no significant publication bias for studies assessing AMH values immediately after chemotherapy (*p* = 0.5 and *p* = 0.3, respectively) and for those assessing AMH values 1 year after chemotherapy (*p* = 0.4 and *p* = 0.1, respectively).

#### 3.4.2. Subgroup Analysis

Considering the high heterogeneity in the overall analysis, subgroup analysis was performed when data were available based on factors that could contribute to heterogeneity between the included studies.

##### Subgroup Analysis: Age

The first subgroup analysis was conducted among patients with a median age of more than 40 years old, from ages 35 to 40 years old and from ages 30 to 35 years old, one year after chemotherapy. We included ten studies. Only one study reported results on women younger than 30 years old [13]; thus, a meta-analysis was not performed.

**Figure 3:**
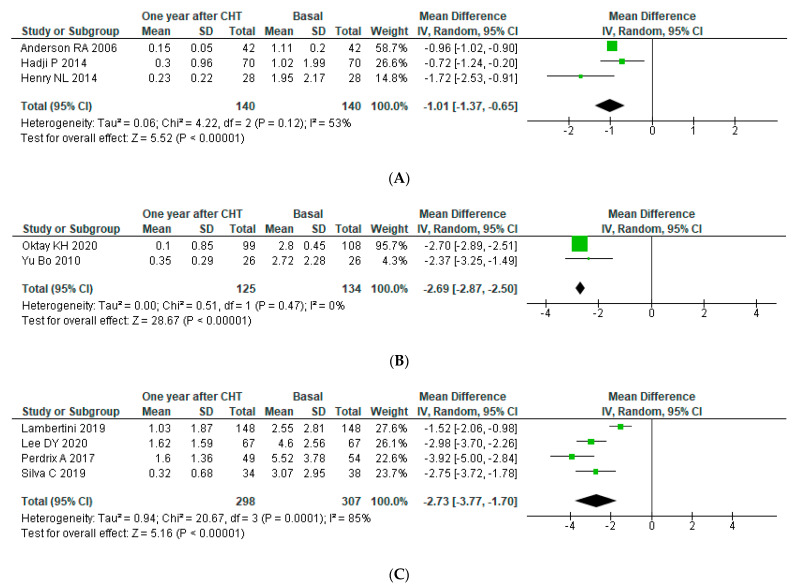
Forest plot for post-operative AMH levels accordingly with age. AMH levels were assessed in women (**A**) older than 40 years, (**B**) between 35 to 40 years, and (**C**) between 30 to 35 years.

An analysis of three studies including 140 patients older than 40 years revealed a statistically significant decline in serum AMH concentration of −1.01 (95% CI: −1.37, −0.65). Two studies (n° of pts = 134) included patients from 35 to 40 years old, reporting a higher impairment (MD −2.69 (95% CI: −2.87, −2.50). Higher toxicity was shown in younger patients (MD −2.73 (95% CI: −3.77, −1.70) aged between 30 and 35 years) assessed in four studies (N = 307). Heterogeneity was acceptable (I^2^ = 53%, I^2^ = 0% and I^2^ = 85%) (See Figure 3).

##### Subgroup Analysis: AMH Assay

**Figure 4:**
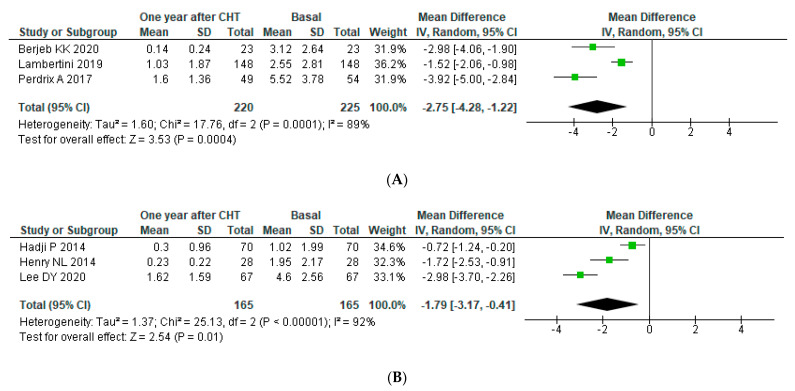
Forest plot for post-operative AMH levels accordingly with AMH assay. AMH levels were assessed with (**A**) ELECSYS kit (**B**) and AMH Gen II Elisa.

We performed a subgroup analysis accordingly with different AMH kits one year after chemotherapy (more data available). A pooled analysis of three studies (N = 225) using Elecsys AMH kits showed a statistically significant decline in serum AMH concentration one year after chemotherapy with an overall reduction of −2.75 (95% CI: −4.28, −1.22) (*p* for heterogeneity < 0.0001, I^2^ = 89%). The level of AMH was higher than that in the analysis of the three studies (*n* = 165) using AMH Gen II kits, which revealed an overall reduction of −1.79 (95% CI: −3–17, −0.41), also with high heterogeneity (*p* for heterogeneity < 0.00001, I^2^ = 92%). Only one study used each of the other four AMH assays (DSL, IOT, pico-AMH and ultrasensitive AMH); therefore, meta-analysis was not performed (See Figure 4).

##### Subgroup Analysis: BRCA Subgroup Analysis

**Figure 5:**
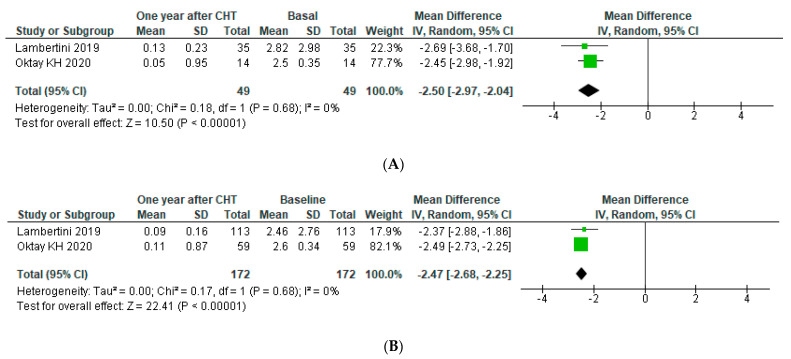
Forest plot for post-operative AMH levels accordingly with BRCA status. AMH levels were assessed at (**A**) m-BRCA (**B**) and wt-BRCA.

A subgroup analysis was performed accordingly with BRCA status one year after chemotherapy (more data available). An analysis of two studies including 49 patients revealed a decline in serum AMH concentration one year after chemotherapy of −2.50 (95% CI: −2.97, −2.04) for BRCA mutated (m-BRCA) patients (p for heterogeneity < 0.00001, I^2^ = 0), similar to the reduction for wild-type BRCA (wt-BRCA) (MD: −2.47 (95% CI: −2.68, −2.25) on 172 patients (*p* for heterogeneity < 0.00001, I^2^ = 0)) (See Figure 5).

## 4. Discussion

This paper is the first systematic review and meta-analysis to evaluate the effect of chemotherapy on AMH in BC patients. The overall analysis revealed a marked decline of AMH value after chemotherapy treatment. AMH levels drop significantly soon after chemotherapy, with some recovery 6 months later. However, combining the detrimental effect of older age and the damage caused by chemotherapy, the ovarian reserve was almost completely depleted for women over 35 years old.

Fertile women represent almost 10% of cases with BC diagnosis [2]. In 2018, to improve the quality of life in cancer survivors [38,39], the American Society of Clinical Oncology suggested a discussion of this reproductive issue and offered fertility preservation strategies before starting cancer treatment [40]. Especially for BC, generally undergoing a polychemotherapy regimen, the risk of ovarian reserve loss is very high [41].

Different authors proposed chemotherapy-induced amenorrhea (CIA) as markers of ovarian reserve to assess the effect of chemotherapy. Women older than 40 years of age had between 77% to 100% risk of developing CIA (77–100%) compared with younger people (0–40%) [42,43,44].

However, amenorrhea is a poor surrogate for fertility because it serves as an instant measure of ovarian function. Above all, resumption of menses could occur after a long time. In addition, pregnancy can also arise during amenorrhea due to sporadic ovulation [45].

In our study, we evaluate chemotherapy-induced gonadotoxicity by AMH levels. In the last 15 years, AMH showed a strong correlation with ovarian reserve, with higher accuracy compared with other markers such as FSH [7].

This metanalysis demonstrated a marked fall in the ovarian reserve after chemotherapy. Although a few months later there was a slight recovery, AMH values remained in the poor responder’s threshold. Therefore, BC women, after ovarian stimulation, will probably obtain a low number of oocytes, resulting in poor chances of becoming pregnant [46].

In our paper, AMH recovers following the first chemotherapy decline, dropping again after 6 months, probably due to physiological aging decline [47]. Previously, Andersen et al. have shown that recovery slope and peak depend on age and type of treatment; in fact, while BC patient’s recovery was slight [10], in other cancers such as lymphoma, for which median age is younger, recovery was greater and longer [48].

The high statistical heterogenicity found in the overall meta-analysis could be due to the high clinical variability, including different ages, chemotherapy regimens, hormone therapies, AMH assay kits and BRCA mutations [49]. In order to reduce heterogeneity, we could perform a sub-group analysis for some of these items, with an overall decrease in heterogeneity that was significant for age and BRCA status and slighter for AMH assay kit.

Instead, given the lack of data, a subgroup analysis for other items, such as chemotherapy regimens, aGnRH treatments and endocrine therapies, remained unaddressed in this meta-analysis. Furthermore, the differences in the methodological quality of the included studies could contribute to the high statistical heterogeneity.

Regarding age, in our study, we showed a different chemotherapy effect accordingly for each age category. Indeed, a lower reduction was shown for older compared with younger women (M D was −1.01, −2.69 and −2.73 for the >40 years, 35 to 40 years and 30 to 35 years subgroups, respectively). The lower baseline level of AMH in older patients probably explains this effect. In fact, through the years, serum AMH levels gradually decline even in healthy women (5.6% per year) [47].

However, the most important finding from this analysis is the AMH value after chemotherapy. It appears to be very low for women older than 35 years of age (mean AMH values one year after CHT for the age category were 0.24 ± 0.69, 0.15 ± 0.77 and 1.14 ± 1.65 ng/mL for the >40 years, 35 to 40 years and 30 to 35 years subgroups, respectively). For these patients, pre-treatment counseling should be mandatory to inform them about the expected fertility drop. Physicians should inform women with pregnancy desire about fertility preservation strategies that could be implemented before starting BC treatment.

Among the 18 included studies, six different AMH assays were used. To reduce bias, standardizing the methodology might be useful in future research, especially since each test reported different sensitivities, detection limits and inter-variability [50]. Indeed, heterogeneity was slightly reduced in the subgroup that used the best performing kit [51].

We also analyzed the impact of CT on AMH levels among BRCA mutation carriers (m-BRCA). Indeed, several studies focused on their fertility potential, since the baseline ovarian reserve is expected to be reduced because of the lack of DNA double-stranded break repair [52]. However, studies on the reduction of AMH levels in the m-BRCA women compared to wild type before cancer treatments are controversial, with opposite results [53].

Moreover, data on the effects of chemotherapy depending on BRCA status are poor. In our sub-analysis, we identified only two studies. Our results suggest that BRCA mutation do not seem to change the effect of chemotherapy on ovarian reserve (MD: −2.50 (95% CI: −2.97, −2.04) versus −2.47 (95% CI: −2.68, −2.25) in mBRCA and wtBRCA, respectively). Further studies are needed to clarify this issue, as the total population in the meta-analysis was small (mBRCA *n* = 172; wtBRCA *n* = 49).

This systematic review and meta-analysis highlighted several gaps in current knowledge and could potentially help guide future research. Indeed, many sub-analyses that would be clinically very helpful and informative cannot be performed with the available data. Many relevant questions remain unsolved: First, what is the possible role of different chemotherapy regimens? Chemotherapy drugs are already known to impact menses in different ways. Anthracycline has a higher risk of amenorrhea than other drugs, but it is not known whether it directly affects ovarian reserve [4].

Second, what are the effects of GnRHa administered during and after chemotherapy on ovarian reserve? No studies in the literature investigate this topic in patients with BC, although several authors have demonstrated that GnRHa could influence AMH levels in healthy women. This question is particularly relevant for BC women since 2015. Based on evidence from the SOFT and TEXT trials, all high-risk patients younger than 35 years of age undergoing chemotherapy are currently receiving ovarian suppression with GnRHa as a standard [54,55].

Third, how do AMH levels vary during adjuvant endocrine therapy? We already know that tamoxifen could induce amenorrhea. Despite this, the effect on AMH remains unknown. There are no data in the literature, but as 75% of women with BC are suitable for endocrine therapy [56], we should clarify the effect of tamoxifen or aromatase inhibitors on ovarian reserve.

The results of this meta-analysis should be interpreted in the light of some limitations, such as publication bias, having included only peer-reviewed, English-language articles; the failure to stratify the results by factors such as chemotherapy regimen, ovarian suppression administration and endocrine therapy because data were unavailable; in general, the low-to-medium methodological quality of the studies were unrepresentative and carried out on small sample sizes, for which no stratified analysis had been performed to contain possible confounding factors.

However, despite the above limitations, this meta-analysis provides a practical tool for predicting ovarian reserve in BC patients undergoing chemotherapy. These findings should be routinely discussed during oncofertility counseling and used to guide fertility preservation choices or even simply to reduce the emotional stress associated with unexpected reproductive health impairment. Future efforts should be made to improve knowledge with more systematically collected data, precisely oriented toward clinical stratification based on the key risk factors.

## Data Availability

Not applicable.

## Supplementary Materials

The following are available online at https://www.mdpi.com/article/10.3390/jpm11080704/s1, Supplementary Material Table S1: Search strategy; Supplementary Material Table S2: Data extraction 1; Supplementary Material Table S3: Data extraction 2; Supplementary Material Table S4: Quality assessment 1; Supplementary Material Table S5: Quality assessment 2.

## References

[1] Sung H., Ferlay J., Siegel R.L., Laversanne M., Soerjomataram I., Jemal A., Bray F. (2021). Global cancer statistics 2020: GLOBOCAN estimates of incidence and mortality worldwide for 36 cancers in 185 countries. CA Cancer J. Clin..

[2] Bardia A., Hurvitz S. (2018). Targeted Therapy for Premenopausal Women with HR+, HER2− Advanced Breast Cancer: Focus on Special Considerations and Latest Advances. Clin. Cancer Res..

[3] Cardoso F., Kyriakides S., Ohno S., Penault-Llorca F., Poortmans P., Rubio I.T., Zackrisson S., Senkus E. (2019). Early breast cancer: ESMO Clinical Practice Guidelines for diagnosis, treatment and follow-up. Ann. Oncol..

[4] Zavos A., Valachis A. (2016). Risk of chemotherapy-induced amenorrhea in patients with breast cancer: A systematic review and meta-analysis. Acta Oncol..

[5] Hansen K.R., Hodnett G.M., Knowlton N., Craig L.B. (2011). Correlation of ovarian reserve tests with histologically determined primordial follicle number. Fertil. Steril..

[6] Alipour F., Rasekhjahromi A., Maalhagh M., Sobhanian S., Hosseinpoor M. (2015). Comparison of Specificity and Sensitivity of AMH and FSH in Diagnosis of Premature Ovarian Failure. Dis. Markers.

[7] Broekmans F.J., Kwee J., Hendriks D.J., Mol B.W., Lambalk C.B. (2006). A systematic review of tests predicting ovarian reserve and IVF outcome. Hum. Reprod. Updat..

[8] Eslami B., Jalaeefar A., Moini A., Omranipour R., Haghighi M., Alipour S. (2020). Significant Post-Chemotherapy Decrease of Ovarian Reserve in Iranian Women With Breast Cancer. Acta Med. Iran..

[9] Al-Rawi S.A., Saleh B.O., Al-Naqqash M. (2018). Serum anti-müllerian hormone levels in evaluation of chemotherapy effect on ovarian reserve in women with breast cancer. Saudi Med. J..

[10] Anderson R.A., Themmen A.P.N., Al-Qahtani A., Groome N.P., Cameron D.A. (2006). The effects of chemotherapy and long-term gonadotrophin suppression on the ovarian reserve in premenopausal women with breast cancer. Hum. Reprod..

[11] Anderson R.A., Cameron D.A. (2011). Pretreatment Serum Anti-Müllerian Hormone Predicts Long-Term Ovarian Function and Bone Mass after Chemotherapy for Early Breast Cancer. J. Clin. Endocrinol. Metab..

[12] Bala J., Seth S., Dhankhar R., Ghalaut V.S. (2016). Chemotherapy: Impact on Anti-Müllerian Hormone Levels in Breast Carcinoma. J. Clin. Diagn. Res..

[13] Berjeb K.K., Debbabi L., Braham M., Zemni Z., Chtourou S., Hannachi H., Hamdoun M., Ayadi M., Kacem K., Zhioua F. (2021). Evaluation of ovarian reserve before and after chemotherapy. J. Gynecol. Obstet. Hum. Reprod..

[14] D’Avila Â.M., Capp E., Corleta H.V.E. (2017). Antral Follicles Count and Anti-Müllerian Hormone Levels after Gonadotoxic Chemotherapy in Patients with Breast Cancer: Cohort Study. Rev. Bras. Ginecol. Obs. RBGO Gynecol. Obstet..

[15] Dezellus A., Barriere P., Campone M., Lemanski C., Vanlemmens L., Mignot L., Delozier T., Levy C., Bendavid C., Debled M. (2017). Prospective evaluation of serum anti-Müllerian hormone dynamics in 250 women of reproductive age treated with chemotherapy for breast cancer. Eur. J. Cancer.

[16] Hadji P., Kauka A., Ziller M., Birkholz K., Baier M., Muth M., Kann P. (2014). Effect of adjuvant endocrine therapy on hormonal levels in premenopausal women with breast cancer: The ProBONE II study. Breast Cancer Res. Treat..

[17] Henry N.L., Xia R., Schott A.F., McConnell D., Banerjee M., Hayes D.F. (2013). Prediction of Postchemotherapy Ovarian Function Using Markers of Ovarian Reserve. Oncology.

[18] Lambertini M., Olympios N., LeQuesne J., Calbrix C., Fontanilles M., Loeb A., Leheurteur M., Demeestere I., Di Fiore F., Perdrix A. (2019). Impact of Taxanes, Endocrine Therapy, and Deleterious Germline BRCA Mutations on Anti-müllerian Hormone Levels in Early Breast Cancer Patients Treated With Anthracycline- and Cyclophosphamide-Based Chemotherapy. Front. Oncol..

[19] Lee D.-Y., Kim J.-Y., Yu J., Kim S.W. (2020). Prediction of Successful Ovarian Protection Using Gonadotropin-Releasing Hormone Agonists During Chemotherapy in Young Estrogen Receptor-Negative Breast Cancer Patients. Front. Oncol..

[20] Oktay K.H., Bedoschi G., Goldfarb S.B., Taylan E., Titus S., Palomaki G.E., Cigler T., Robson M., Dickler M.N. (2020). Increased chemotherapy-induced ovarian reserve loss in women with germline BRCA mutations due to oocyte deoxyribonucleic acid double strand break repair deficiency. Fertil. Steril..

[21] Passildas J., Collard O., Savoye A.-M., Dohou J., Ginzac A., Thivat E., Durando X., Kwiatkowski F., Penault-Llorca F., Abrial C. (2019). Impact of Chemotherapy-induced Menopause in Women of Childbearing Age With Non-metastatic Breast Cancer—Preliminary Results From the MENOCOR Study. Clin. Breast Cancer.

[22] Perdrix A., Saint-Ghislain M., Degremont M., David M., Khaznadar Z., Loeb A., Leheurteur M., Di Fiore F., Clatot F. (2017). Influence of adjuvant chemotherapy on anti-Müllerian hormone in women below 35 years treated for early breast cancer. Reprod. Biomed. Online.

[23] Silva C., Rama A.C.R., Soares S.R., Moura-Ramos M., Santos T.A. (2019). Adverse reproductive health outcomes in a cohort of young women with breast cancer exposed to systemic treatments. J. Ovarian Res..

[24] Trapp E., Steidl J., Rack B., Kupka M., Andergassen U., Jückstock J., Kurt A., Vilsmaier T., de Gregorio A., Tzschaschel M. (2017). Anti-Müllerian hormone (AMH) levels in premenopausal breast cancer patients treated with taxane-based adjuvant chemotherapy—A translational research project of the SUCCESS A study. Breast.

[25] Yu B., Douglas N., Ferin M.J., Nakhuda G.S., Crew K., Lobo R.A., Hershman D.L. (2010). Changes in markers of ovarian reserve and endocrine function in young women with breast cancer undergoing adjuvant chemotherapy. Cancer.

[26] Page M.J., McKenzie J.E., Bossuyt P.M., Boutron I., Hoffmann T.C., Mulrow C.D., Shamseer L., Tetzlaff J.M., Akl E.A., Brennan S.E. (2021). The PRISMA 2020 statement: An updated guideline for reporting systematic reviews. BMJ.

[27] Ouzzani M., Hammady H., Fedorowicz Z., Elmagarmid A. (2016). Rayyan—a web and mobile app for systematic reviews. Syst. Rev..

[28] Wan X., Wang W., Liu J., Tong T. (2014). Estimating the sample mean and standard deviation from the sample size, median, range and/or interquartile range. BMC Med. Res. Methodol..

[29] Higgins J.P.T., Thompson S.G., Deeks J.J., Altman D.G. (2003). Measuring inconsistency in meta-analyses. BMJ.

[30] Egger M., Smith G.D., Schneider M., Minder C. (1997). Bias in meta-analysis detected by a simple, graphical test. BMJ.

[31] Begg C.B., Mazumdar M. (1994). Operating Characteristics of a Rank Correlation Test for Publication Bias. Biometrics.

[32] Galbraith R.F. (1988). A note on graphical presentation of estimated odds ratios from several clinical trials. Stat. Med..

[33] Anderson R., Mansi J., Coleman R., Adamson D., Leonard R. (2017). The utility of anti-Müllerian hormone in the diagnosis and prediction of loss of ovarian function following chemotherapy for early breast cancer. Eur. J. Cancer.

[34] Wakimoto Y., Fukui A., Wakimoto G., Ikezawa Y., Matsuoka M., Omote M., Sugiyama Y., Ukita Y., Kato T., Shibahara H. (2019). Association between spontaneous ovulation and serum anti-Müllerian hormone levels in a premature ovarian insufficiency patient after a multimodal treatment for breast cancer. J. Obstet. Gynaecol. Res..

[35] Mahany E.B., Hershman D.L., Sauer M.V., Choi J.M. (2013). Pregnancy despite ovarian insufficiency in a patient with breast cancer. Reprod. Med. Biol..

[36] Miyoshi Y., Ohta H., Namba N., Tachibana M., Miyamura T., Miyashita E., Hashii Y., Oue T., Isobe A., Tsutsui T. (2013). Low Serum Concentrations of Anti-Müllerian Hormone Are Common in 53 Female Childhood Cancer Survivors. Horm. Res. Paediatr..

[37] Andergassen U., Kasprowicz N.S., Hepp P., Schindlbeck C., Harbeck N., Kiechle M., Sommer H., Beckmann M.W., Friese K., Janni W. (2013). Participation in the SUCCESS-A Trial Improves Intensity and Quality of Care for Patients with Primary Breast Cancer. Geburtshilfe Frauenheilkd..

[38] Benedict C., Thom B., Friedman D.N., Pottenger E., Raghunathan N., Kelvin J.F. (2018). Fertility information needs and concerns post-treatment contribute to lowered quality of life among young adult female cancer survivors. Support. Care Cancer.

[39] Gorman J.R., Su H.I., Mph S.C.R., Dominick S.A., Malcarne V. (2015). Experiencing reproductive concerns as a female cancer survivor is associated with depression. Cancer.

[40] Oktay K., Harvey B., Partridge A.H., Quinn G., Reinecke J., Taylor H.S., Wallace W.H., Wang E.T., Loren A.W. (2018). Fertility Preservation in Patients With Cancer: ASCO Clinical Practice Guideline Update. J. Clin. Oncol..

[41] Early Breast Cancer Trialists’ Collaborative Group (EBCTCG) (2005). Effects of chemotherapy and hormonal therapy for early breast cancer on recurrence and 15-year survival: An overview of the randomised trials. Lancet.

[42] Smith I.E., Dowsett M., Yap Y.-S., Walsh G., Lonning P.E., Santen R.J., Hayes D. (2006). Adjuvant Aromatase Inhibitors for Early Breast Cancer After Chemotherapy-Induced Amenorrhoea: Caution and Suggested Guidelines. J. Clin. Oncol..

[43] Davis A.L., Klitus M., Mintzer D.M. (2005). Chemotherapy-Induced Amenorrhea from Adjuvant Breast Cancer Treatment: The Effect of the Addition of Taxanes. Clin. Breast Cancer.

[44] Walshe J.M., Denduluri N., Swain S.M. (2006). Amenorrhea in Premenopausal Women After Adjuvant Chemotherapy for Breast Cancer. J. Clin. Oncol..

[45] Van Der Wijden C., Manion C. (2015). Lactational amenorrhoea method for family planning. Cochrane Database Syst. Rev..

[46] Polyzos N.P., Drakopoulos P., Parra J., Pellicer A., Santos-Ribeiro S., Tournaye H., Bosch E., Garcia-Velasco J. (2018). Cumulative live birth rates according to the number of oocytes retrieved after the first ovarian stimulation for in vitro fertilization/intracytoplasmic sperm injection: A multicenter multinational analysis including ∼15,000 women. Fertil. Steril..

[47] Api M. (2009). Is ovarian reserve diminished after laparoscopic ovarian drilling?. Gynecol. Endocrinolol..

[48] Decanter C., Morschhauser F., Pigny P., Lefebvre C., Gallo C., Dewailly D. (2010). Anti-Müllerian hormone follow-up in young women treated by chemotherapy for lymphoma: Preliminary results. Reprod. Biomed. Online.

[49] Higgins J., Thompson S., Deeks J., Altman D. (2002). Statistical heterogeneity in systematic reviews of clinical trials: A critical appraisal of guidelines and practice. J. Heal. Serv. Res. Policy.

[50] Su H.I., Sammel M.D., Homer M.V., Bui K., Haunschild C., Stanczyk F.Z. (2014). Comparability of antimüllerian hormone levels among commercially available immunoassays. Fertil. Steril..

[51] Gassner D., Jung R. (2014). First fully automated immunoassay for anti-Müllerian hormone. Clin. Chem. Lab. Med..

[52] Venkitaraman A.R. (2002). Cancer Susceptibility and the Functions of BRCA1 and BRCA2. Cell.

[53] Corrado G., Marchetti C., Trozzi R., Scambia G., Fagotti A. (2021). Fertility preservation in patients with BRCA mutations or Lynch syndrome. Int. J. Gynecol. Cancer.

[54] Francis P., Regan M.M., Fleming G.F., Láng I., Ciruelos E., Bellet M., Bonnefoi H.R., Climent M.A., Da Prada G.A., Burstein H.J. (2015). Adjuvant Ovarian Suppression in Premenopausal Breast Cancer. N. Engl. J. Med..

[55] Regan M.M., Pagani O., Fleming G.F., Walley B.A., Price K.N., Rabaglio M., Maibach R., Ruepp B., Coates A.S., Goldhirsch A. (2013). Adjuvant treatment of premenopausal women with endocrine-responsive early breast cancer: Design of the TEXT and SOFT trials. Breast.

[56] Kennecke H., Yerushalmi R., Woods R., Cheang M.C.U., Voduc D., Speers C.H., Nielsen T.O., Gelmon K. (2010). Metastatic Behavior of Breast Cancer Subtypes. J. Clin. Oncol..

