# Supplementary material for: Ovarian Reserve after Chemotherapy in Breast Cancer: A Systematic Review and Meta-Analysis
Source: J Pers Med. 2021 Jul 23;11(8):704. doi: 10.3390/jpm11080704 (PMC8400427; doi:10.3390/jpm11080704)
Supplement: Supplementary file 1 [file jpm-11-00704-s001.zip › Table S1.pdf]

Supplementary Materials

Table S1. Search strategy.

PUBMED string
(("Anti-Mullerian Hormone"[Mesh] OR "Antimullerian hormone" OR "anti mullerian hormone" OR "anti-mullerian hormone" OR "Mullerian-Inhibiting Factor" OR "Anti-Mullerian Factor" OR "Mullerian Inhibitory Substance" OR "Mullerian Inhibiting Hormone" OR AMH OR "Ovarian Reserve"[Mesh])<br>AND (("Breast Neoplasms"[Mesh] OR "Breast Neoplasm*" OR "Breast Tumor*" OR "Mammary Cancer*" OR "breast cancer*" OR "breast carcinoma*" OR "mammary carcinoma" OR breast malignanc* OR "Breast Malignant Neoplasm*" OR "Breast Malignant Tumor")<br>AND (chemotherapy OR chemotherapies OR "Cancer Chemotherapy Agent*" OR "Cancer Chemotherapy Drug*" OR "Chemotherapeutic Anticancer Agent*" OR "chemotherapeutic agent*")))
SCOPUS string
(("Anti-Mullerian Hormone"[Mesh] OR "Antimullerian hormone" OR "anti mullerian hormone" OR "anti-mullerian hormone" OR "Mullerian-Inhibiting Factor" OR "Anti-Mullerian Factor" OR "Mullerian Inhibitory Substance" OR "Mullerian Inhibiting Hormone" OR AMH OR "Ovarian Reserve"[Mesh])<br>AND (("Breast Neoplasms"[Mesh] OR "Breast Neoplasm*" OR "Breast Tumor*" OR "Mammary Cancer*" OR "breast cancer*" OR "breast carcinoma*" OR "mammary carcinoma" OR breast malignanc* OR "Breast Malignant Neoplasm*" OR "Breast Malignant Tumor")<br>AND (chemotherapy OR chemotherapies OR "Cancer Chemotherapy Agent*" OR "Cancer Chemotherapy Drug*" OR "Chemotherapeutic Anticancer Agent*" OR "chemotherapeutic agent*")))
728 articles
