# Supplementary material for: Ovarian Reserve after Chemotherapy in Breast Cancer: A Systematic Review and Meta-Analysis
Source: J Pers Med. 2021 Jul 23;11(8):704. doi: 10.3390/jpm11080704 (PMC8400427; doi:10.3390/jpm11080704)
Supplement: Supplementary file 1 [file jpm-11-00704-s001.zip › Table S2.pdf]

Supplementary Materials

Table S2. Data Extraction 1.

First Author, Year	Country	Study Design	Enrolment	Sample size	Age (years)	Chemiotherapy regimen (cycles)	aGnRH during chemotherapy	Adjuvant aGnRH	Hormone Therapy
**Lambertini, 2019**	FR	RC	2008-2016	148	Mdn, [IQR] 35 [31.5–38]	6-FEC or<br>3-FEC+3-D	No	Yes	Tam
**Perdrix, 2017**	FR	RC	2008-2014	54	Mdn [Ra] 31.5 [11–35]	6-FEC or<br>3-FEC+3-D	No	No	Tam
**Oktay, 2020**	US	PC	2009-	108	M [SD] 35.8 [4.0]	AC-T, CFM or EC-T	N/A	N/A	N/A
**Lee, 2020**	KR	PC	2013-2017	67	M [SD] 33.2 [3.6]	4-AC,<br>4-AC-T or<br>6-CAF	Goserelin or leuplin: before starting chemotherapy and every 4 weeks thereafter until completion of chemotherapy	No	No
**Shin, 2020**	KR	PC	2009-2016	136	Mdn [Ra] 32 [19-39]	6- CAF, 4-AC-T, 4-AC-D2 or others including 6 CMFa	GnRHa at least 1 day before the chemotherapy until at least once after the last dose of chemotherapy. Subject were divided into 3 groups according to the interval between start on GnRH and the start of chemotherapy: 1-6 days, 7-13 days, and ≥ 14 days. The mean of administration is 158.6 days.	No	Tam (95.3%)
**Berjeb, 2020**	TN	RC	2015-2018	23	M [SD] 26.7 [6.8]	FEC-D	N/A	N/A	N/A
**Eslami, 2020**	IR	PC	2018-2020	46	M [SD] (Ra) 26.7 [4.58] (25-45)	N/A	N/A	N/A	N/A
**Silva, 2019**	PT	PC	2014-2016	38	M [Mdn] (min-max) 32.9	FEC or AC or EC-T	N/A	Yes	n = 25 Tam = 7 (28%) Tam +GnRHa=

					[33] (25-39)				10 (40%)AI=1 (4%), AI+ GnRHa= 5 (20%)GnRHa= 2 (8%)
**Passildas, 2019**	FR	PC	2014-2015	58	Mdn [Ra] 40.8 [24.7-46]	3- or 4-FEC + 3- or 4-Ta, 4–6-TAC, 4 AC-T or TCH	N/A	N/A	N/A
**Al-Rawi, 2018**	IQ	PC	2016-2017	30	M [SE] 38.83 [4.74]	4-AC	N/A	N/A	N/A
**Trapp, 2017**	DE	RCT	2005-2007	179	Mdn [Ra] 36 [21-40]	3- FEC-D or 3- FEC-D2+ Gem	Ovarian protection during chemotherapy 7.1%	Tam 20 mg/day + goserelin 3.6 mg s.c.q4w (52.9%) or goserelin alone 3.6 mg s.c.q4w (0.6%)	Tam 20 mg/day (8.8%), Tam 20 mg/day + goserelin 3.6 mg s.c.q4w (52.9%), or goserelin alone 3.6 mg s.c.q4w (0.6%)
**D'Avila, 2017**	BR	PC	2007-2009	52	M [SD] (Ra) 35.3 [3.8] (27-40)	N/A	N/A	N/A	N/A
**Dezelleus, 2017**	FR	PC	2010-2011	250	M [SD] 34.8 [3.9]	3-FEC-D (88.8%), 3-FEC (4.8%), AC-T (0.4%), adriblastin and taxane (0.4%), or taxane alone (5.6%)	3.6%	No	Tam (60.2%)
**Henry, 2014**	US	PC	2007-2008	28	Mdn [Ra] 41 [25-50]	Dosedense doxorubicin and cyclophosphamide (32.1%), dosedense doxorubicin and cyclophosphamide (32.1%), weekly paclitaxel (25%), docetaxel/cyclophosphamide (32.1%) and docetaxel/carboplatin (3.6%)	N/A	AI+ GnRHa (21.4%)	Tam (50%), AI or +GnRHa (21.4%)
**Hadji, 2014**	DE	RCT	2005-2009	70	M [Ra] 41 [25-50]	Anthracycline-cyclophosphamide followed by a taxane and fluorouracil	N/A	GnRH analogues (84.3%)	GnRH a (84.3%), Tam (94%) or ZOL (n=34)
**Yu, 2010**	US	RCT	2002-2006	26	Mdn [Ra] 37 [27-40]	AC-T, CMF or CAF	No	No	Tam or AI
**Anderson, 2006/2011**	UK	PC	2001-2003	42	Mdn [Ra] 41 [28.6-52.7]	AC, CMF or Adriamycin followed by CMF or Epirubicin followed by CMF, FEC-D or EC-T (Gem)	No	No	No
**Bala, 2016**	IN	PCC	2013-2014	60	N/A	4- FEC-D	N/A	N/A	N/A

Abbreviations

RC = retrospective cohort

PC = prospective cohort

RCT = randomized controlled trial

PCC = prospective case-control

Mdn = median

M = mean

IQR = interquartile

Ra = range

SD = standard deviation

SE = standard error

Chemotherapy regimen

FEC = five fluorouracil 500 mg/m2, epirubicin 100 mg/m2 and cyclophosphamide 500 mg/m2

D = docetaxel 100 mg/m2

D2 = docetaxel 75 mg/m2

AC-T = doxorubicin plus cyclophosphamide followed by paclitaxel

CMF = cyclophosphamide plus methotrexate plus fluorouracil

EC-T = epirubicin plus cyclophosphamide followed by docetaxel

AC = doxorubicin (60 mg/m2) plus cyclophosphamide (600 mg/m2)

T = paclitaxel (175 mg/m2)

CAF = cyclophosphamide (500 mg/m2) plus doxorubicin (50 mg/m2) plus 5-fluorouracil (500 mg/m2)

CMFa = cyclophosphamide (50 mg per oral thrice a day for 14 days) plus methotrexate (40 mg/m2 on days 1 and 8) plus 5-fluorouracil (600 mg/m2 on days 1 and 8)

FEC-D = 5-fluorouracil, epirubicin and cyclophosphamide plus docetaxel

Ta = Taxotere

TAC = docetaxel, adriamycin and cyclophosphamide

TCH = docetaxel/paclitaxel, carboplatin and herceptin

Gem = gemcitabine

Tam = tamoxifene

AI = aromatase inhibitors

ZOL = zoledronic acid

N/A = not available
