# Supplementary material for: Ovarian Reserve after Chemotherapy in Breast Cancer: A Systematic Review and Meta-Analysis
Source: J Pers Med. 2021 Jul 23;11(8):704. doi: 10.3390/jpm11080704 (PMC8400427; doi:10.3390/jpm11080704)
Supplement: Supplementary file 1 [file jpm-11-00704-s001.zip › Table S3.pdf]

Supplementary Materials

Table S3. Data Extraction 2.

First Author, Year	Assay AMH	Baseline AMH	Follow-up	1-year AMH	3-year AMH	5-year AMH	Subgroup Analysis of AMH measurements before and after Chemotherapy
**Lambertini, 2019**	Elecsys	M [SD]:<br>2.55ng/mL<br>[2.81]	Mdn [IQR]days:<br>1132 [1099-1186]	M [SD]:<br>0.207ng/ml [0.277]	M [SD]:<br>0.103 ng/ml [0.187]	N/A	***Chemotherapy Regimen***:<br>Baseline AMH (M [SD])<br>▪ FEC: 2.39 [1.88], FEC-D: 2.57 [2.94].<br>1 year-AMH (M [SD])<br>▪ FEC: 0.22 [0.1-0.34], FEC-D: 0.04 [0-0.2].<br>3 years- AMH (M [SD])<br>▪ FEC: 0.06 [0-0.2], FEC-D: 0.18[0-0.35].<br>***Hormone therapy (HT):***<br>Baseline AMH (M [SD])<br>▪ HT:2.88 [3.30], No-HT: 2.03 [1.68].<br>1 year-AMH (M [SD])<br>▪ HT: 0.12 [0-0.2], No-HT: 0.02 [0-0.1].<br>3 years- AMH (M [SD])<br>▪ HT: 0.11[0-0.3], No-HT: 0.2[0-0.3].<br>***BRCA mutational status:***<br>Baseline AMH (M [SD])<br>▪ BRCA+: 2.82 [2.98], BRCA-: 2.46 [2.76].<br>1 year-AMH (M [SD])<br>▪ BRCA+: 0.09 [0-0.15], BRCA-: 0.06[0-0.12].<br>3 years- AMH (M [SD])<br>▪ BRCA+: 0.25[0-1.15], BRCA-: 0.16[0-0.3].

**Perdrix, 2017**	Elecsys	Mdn [Ra]:<br><br>2.12 ng/ml [0.33–17.49]	Mdn [IQR]days:<br><br>1913[1770–1913]	Mdn [min-max]:<br><br>0.13 ng/ml [0.01–6.11]<br><br>n=49	Mdn [min-max]:<br><br>0.31 ng/ml [0.01–3.28]<br><br>n=32	Mdn:0.29 ng/ml n=9	***Age:***<br><br>Baseline AMH (Mdn [Ra])<br><br>▪ 20-24 years old (n=2): 3.37 ng/mL (3.09-3.65),<br>25-29 years old (n=10): 2.29 ng/mL (1.18-17.49), 30-35 years old (n=42): 1.95 ng/mL (0.34-7.2).<br>***Smoke:***<br><br>Baseline AMH (Mdn [Ra])<br><br>▪ Smokers (n=16): 2.54 ng/mL (0.83-6.5),<br>No-smokers (n=38):1.95 ng/mL (0.37-17.49).<br>***BRCA mutational status:***<br><br>Baseline AMH (Mdn [Ra])<br><br>▪ BRCA+ (n=14): 2.13 ng/mL (0.33-6.50),<br>▪ BRCA- (n=40): 2.12 ng/mL (0.57-17.49).<br>***Body mass Index kg/m2 (BMI):***<br><br>Baseline AMH (Mdn [Ra])<br><br>▪ BMI>25 (n=14): 3.29 ng/mL (0.78-17.49),<br>BMI<25 (n=40): 1.82 ng/mL (0.33-7.2).<br>***Pregnancy status:***<br><br>Baseline AMH (Mdn [Ra])<br><br>▪ Pregnancy: 2.00 ng/mL (0.33-17.49),<br>No-Pregnancy: 2.25 ng/mL (0.56-5.13).<br>***Chemotherapy Regimen***:<br><br>1 year-AMH (Mdn [Ra])<br><br>▪ 3FEC-3D (n=41): 0.09 ng/mL (0.01-6.11),<br>6FEC (n=8): 0.39 ng/mL (0.21-2.33).<br>***Hormone therapy (HT):***<br><br>3 year-AMH (Mdn [Ra])

							▪ HT (n=15): 0.34 ng/mL (0.01-2.76), No-HT (n=17): 0.25 ng/mL (0.01-3.28).
**Oktay, 2020**	picoAMH	M [SD]:<br>2.8 ng/mL [0.45]	24 months	M [SD]:<br>0.098 ng/ml [0.85]<br>n=99 (12 months)<br><br>M [SD]:<br>0.10 ng/ml [0.91]<br>n=86 (18 months)	M [SD]:<br>0.15 ng/ml [0.86]<br>n=73 (24 months)	N/A	***BRCA mutational status:***<br>Baseline AMH (M [SD])<br>▪ BRCA not tested (n=35):3.4 ng/mL (0.31), BRCA- (n=59): 2.6 ng/mL (0.31), BRCA+ (n=14): 2.5 ng/mL (0.35).<br>1 year-AMH (M [SD])<br>▪ BRCA not tested (n=33): 0.10 ng/mL (0.78), BRCA- (n=52): 0.11 ng/mL (0.91), BRCA+ (n=14): 0.053 ng/mL (0.95).<br>18 months-AMH (M [SD])<br>▪ BRCA not tested (n=30): 0.11 ng/mL (0.91), BRCA- (n=47): 0.12 ng/mL (0.88), BRCA+ (n=9): .029 ng/mL (0.99).<br>24 months-AMH (M [SD])<br>▪ BRCA not tested (n=24): 0.20 ng/mL (0.83), BRCA- (n=41): 0.16 ng/mL (0.86), BRCA+ (n=8): 0.045 ng/mL (0.90).
**Lee, 2020**	Gen II	M [SD]<br>4.6 ng/mL [2.5]	12 months	M: 1.6 ng/mL			
**Shin, 2020**	Gen II	M [SE]:<br>5.0 ng/mL [0.4](1-6 days), 5.3 ng/mL [0.7](7-13 days),	36 months	M [SE]*:<br>3 months* (n=127):<br>4.4 [0.3](1-6 days),<br>5.5 [1.1](7-13 days),<br>3.4 [1.2](>=14 days).	M[SE] *:<br>24 months* (n=73):<br>26.1 [5.3](1-6 days),<br>28[5.2](7-13 days),<br>33.7[7.5](>=14 days).		

		8.1 ng/mL [1.3](>=14 days)		6 months* (n=115):<br>6.9 [6.6](1-6 days),<br>13.1 [3.2](7-13 days),<br>10.0 [3.3](>=14 days).<br><br>12 months* (n=95):<br>22.7 [4.5](1-6 days),<br>29.7 [6.2](7-13 days),<br>32.8 [10.1](>=14 days).	36 months* (n=35):<br>34.7[15.6](1-6 days),<br>30.9[8.0](7-13 days),<br>24.9[8.2](>=14 days).		
**Berjeb, 2020**	Elecsys	M [SD]:<br>3.12 ng/mL [2.64]	M [SD] (Ra)months:<br>16.8 [9.3] (6-41)	M [SD]:<br>0.14 ng/mL [0.24]			
**Eslami, 2020**	Gen II	M [SD] (Mdn) 3.20 ng/mL [2.84] (2.28)	1 month	M [SD](Mdn):<br>0.26 ng/mL [070] (0.11)<br>(value after chemotherapy)			
**Silva, 2019**	Ultrasensitive AMH	M [SD]<br>(Mdn)<br>3.07 ng/mL [2.95] (2.20)	M [Ra]months:<br>18 [6-35]	M [SD](Mdn)<br>0.15 ng/mL [0.46] (0.06)<br>(value after chemotherapy)			
**Passildas, 2019**	Elecsys	Mdn [Ra]:	1 month	Mdn[Ra]:			

		CIM-group: 3.93 pmol/L [0.1-58.1] (n=41)<br><br>Non-CIM group:<br>13.5 pmol/L [1.64-69] (n=17)		CIM-group:<br>0.1 pmol/L [0-0] (n=39)<br><br>Non-CIM group:<br>0.1pmol/L [0.1-1.21] (n=15)			
**Al-Rawi, 2018**	N/A	M [SD]:<br>2.05 ng/mL [0.28]	1 month	M [SE]:<br>0.06ng/mL [0.01]			
**Trapp, 2017**	Gen II	Mdn [SD] (Ra):<br>1.37 ng/mL [2.12]<br>(<0.1-11.3)	2 years	Mdn [SD] (range):<br><0.1 ng/mL [0.01] (<0.1-0.21)<br>(value 4 week after chemotherapy)	Mdn [SD] (Ra):<br><0.1 ng/mL [0.46] (<0.1-3.9).<br>(Only 4 patients AMH level >1ng/ml)		*Age:*<br>Baseline AMH (Mdn [SD](Ra))<br>▪ <35 years old(n=71):1.76 ng/mL [2.02] (<0.1-8.28), >35 years old (n=99):1.21 ng/mL [2.15] (<0.1-11.32)<br>4 weeks-AMH (M)<br>▪ <35 years old: 0.1 ng/mL >35 years old :0.1 ng/mL<br>2 years-AMH (M[SD](Ra))<br>▪ <35 years old: 0.3 ng/mL [0.65] (<0.1-3.9), >35 years old: 0.17 ng/mL [0.27] (<0.1-1.62)
**D'Avila, 2017**	IOT	Mdn [IQR]: 2.53 [1.0-5.31]	M(SD)months:<br>14(3)	Mdn [IQR]:<br><0.08 ng/mL			

				(value at 2 moths)<br>Mdn [IQR]:<br><0.08 ng/mL [<0.08-1.07]<br>(value at 6 months)			
**Dezelleus, 2017**	IOT	M [SD] (Mdn):<br>4.19 ng/mL [4.84] (2.95)	24 months	M [SE]:<br>0.68ng/mL [3.01]<br>(value after chemotherapy)	M [SE]:<br>0.78ng/mL [1.40]		
**Henry, 2014**	Gen II	M [SD]:<br>1.95 [2.17] ng/mL	M [SD]months:<br>13.6 [1.1]	M[SD]: <0.16 ng/mL<br>(value after 1 month)<br>M[SD]: 0.23 ng/mL [0.22]<br>(value after 1 year)			
**Hadji, 2014**	Gen II	M [SD]:<br>0.96 [1.99] mg/mL (placebo group),<br>1.09 [2.02] mg/mL<br>(ZOL group)	12 months	M [SD]:<br>0.41 [1.24] mg/mL (placebo group),<br>0.33 [0.62] mg/mL (ZOL group)<br>(value after 6 months)<br>M [SD]:<br>0.38 [1.31] mg/mL (placebo group),<br>0.22 [0.30] mg/mL (ZOL group)			

				(value after 12 months)			
**Yu, 2010**	DSL	Mdn [Ra]: 0.86 ng/mL [0.07-9.1]	week 52	Mdn [Ra] Week 6: 0.08 ng/mL [<0.05-0-21] ng/mL, Week 12: 0.05 ng/mL [<0.05-0.07], Week 36: 0.05 ng/mL [<0.05-1.7], Week 52: 0.07 ng/mL [<0.05-1.16]			
**Anderson, 2006/2011**	IOT	M [SD]: 1.11 [0.20] ng/mL (2006) M [SD]: 1.29 [0.21] ng/mL (2011)	5 years	M [SE]: 0.15ng/mL [0.05], (value after 1 year) M [SE]: 0.32ng/mL [0.07] (value after 2 years)	M [SE]: 0.14ng/mL [0.04], (value after 3 years) M [SE]: 0.10ng/mL [0.03], (value after 4 years)	M [SE]: 0.09 ng/mL [0.02] (value after 5 years)	
**Bala, 2016**	Gen II	M [SD]: 1.67 [0.44] ng/ml (Experimental Group) 1.9 [0.37] ng/ml	1 month	M [SD]: 1.03 ng/mL [0.25] (value after chemotherapy)			

		(Control Group)					

Abbreviations

Mdn = median

M = mean

IQR = interquartile

Ra = range

SD = standard deviation

SE = standard error

Chemotherapy regimen

FEC = five fluorouracil 500 mg/m2, epirubicin 100 mg/m2 and cyclophosphamide 500 mg/m2

D = docetaxel 100 mg/m2

FEC-D = 5-fluorouracil, epirubicin and cyclophosphamide plus docetaxel

ZOL = zoledronic acid

N/A = not available

Chemotherapy-induced menopausa = CIM-group

No chemotherapy-induced menopausa = Non-CIM group

*AMH value was expressed as pcAMH = (pcAMH value (ng/mL)/baseline AMH value (ng/dL)) x 100 (%).
