# Supplementary material for: Ovarian Reserve after Chemotherapy in Breast Cancer: A Systematic Review and Meta-Analysis
Source: J Pers Med. 2021 Jul 23;11(8):704. doi: 10.3390/jpm11080704 (PMC8400427; doi:10.3390/jpm11080704)
Supplement: Supplementary file 1 [file jpm-11-00704-s001.zip › Table S4-S5.pdf]

Supplementary Materials

Table S4. Quality assessment 1.

Cohort studies NOS

		**OktayKH et al, 2020**	**Lee DY et al, 2020**	**Berjeb JJ et al, 2020**	**Eslami B et al, 2020**	**Lambertini et al, 2019**	**Silva C et al, 2019**	**Passildas J et al, 2019**	**Al Rawi SA et al, 2018**	**Perdrix A et al, 2017**	**D'Avila AM et al, 2017**	**Dezellus A et al, 2017**	**Henry NL et al, 2014**	**Anderson RA et al, 2006**	**Anderson RA et al, 2011**	**Bala J et al, 2016**
**1. Representativeness of the exposed cohort**	a) Truly representative of the average in the target population * (all subjects or random sampling)															
	b) somewhat representative of the average in the target population* (non-	✓				✓						✓				

	random sampling)															
	c) selected group of users		✓	✓	✓		✓	✓	✓	✓	✓		✓	✓	✓	✓
	d) no description of the derivation of the cohort															
**2. Selection of the non-exposed cohort**	a) drawn from the same community as the exposed cohort*															
	b) drawn from a different source															
	c) no description of the derivation of the non-exposed cohort	✓	✓	✓	✓	✓	✓	✓	✓	✓	✓	✓	✓	✓	✓	✓
**3. Ascertainment of**	a) secure	✓	✓	✓	✓	✓	✓	✓	✓	✓	✓	✓	✓	✓	✓	✓

**exposure**	record (e.g., surgical records) *															
	b) structured interview *															
	c) written self-report															
	d) no description															
**4) Demonstration that the outcome of interest was not present at the start of the study**	a) yes *	✓	✓	✓	✓	✓	✓	✓	✓	✓	✓	✓	✓	✓	✓	✓
	b) no															
**1) The subjects in different outcome groups are comparable, based on the study design or analysis. Confounding factors are controlled.**	a) The study controls for the most important factor (age). *	✓	✓	✓												
	b) The study control															

	for any additional factor. *															
**1) Assessment of the outcome**	a) independent blind assessment *															
	b) record linkage *	✓	✓	✓	✓	✓	✓	✓	✓	✓	✓	✓	✓	✓	✓	✓
	c) self-report															
	d) no description															
**2) Was follow-up long enough for outcomes to occur**	a) yes (select an adequate follow up peri od for outcome of interest) *	✓	✓	✓		✓	✓			✓		✓	✓	✓	✓	
	b) no				✓			✓	✓		✓					
**3) Adequacy of follow-up of cohorts**	a) complete follow-up - all subjects accounte		✓	✓	✓	✓		✓	✓					✓	✓	

	d for *															
	b) subjects lost to follow-up unlikely to introduce bias—small number lost to follow-up, or description of those lost provided) *	✓					✓			✓	✓	✓	✓			
	c) low follow-up rate and no description of those lost															✓
	d) no statement															
	Overall quality score	7	6	6	4	6	5	4	4	5	4	6	5	5	5	3

Table S5. Quality assessment 2.

RTC JADAD

**First Author, Year**	**Hadji P et al, 2014**	**Trapp, 2017**	**Yu B et al, 2010**
**1. Was the study described as random?***	Yes	Yes	Yes
**2. Was the randomization scheme described and appropriate?***	No	No	No
**3. Was the study described as double-blind?***	Yes	No	Yes
**4. Was the method of double blinding appropriate? *** *Were both the patient and the assessor appropriately blinded*	No	No	No
**5. Was there a description of dropouts and withdrawals? ***	Yes	Yes	Yes
**Overall quality score**	3—Medium quality	2—Low quality	3—Medium quality
